# Role of epiregulin levels in polycystic ovary syndrome patients: new findings and clinical significance

**DOI:** 10.1590/1806-9282.20242048

**Published:** 2025-07-07

**Authors:** Senay Topsakal, Fatih Altıntas, Guzin Fidan Yaylali, Semin Melahat Fenkci, Sadettin Caliskan

**Affiliations:** 1 Pamukkale University, Faculty of Medicine, Department of Endocrinology and Metabolism – Denizli, Türkiye.; 2 Pamukkale University, Faculty of Medicine, Department of Physiology – Denizli, Türkiye.; 3 Üsküdar University, Faculty of Medicine, Department of Physiology – İstanbul, Türkiye.

**Keywords:** Polycystic ovary syndrome, Epiregulin, Blood

## Abstract

**OBJECTIVE:**

Polycystic ovary syndrome, a prevalent endocrine disorder in reproductive-age women, is characterized by polycystic ovaries, oligoanovulation, and hyperandrogenemia. Epiregulin is a member of the epidermal growth factor (EGF) family, playing a crucial role in processes such as cell growth, differentiation, and proliferation. It is also known to be involved in reproductive system-specific mechanisms, including ovulation and oocyte maturation. This study aimed to investigate the role of epiregulin, an EGF family member implicated in ovulation and oocyte maturation, in women with polycystic ovary syndrome.

**METHODS:**

Serum epiregulin levels were analyzed in 39 women with polycystic ovary syndrome and 28 healthy controls. Associations between epiregulin and clinical/metabolic parameters such as body mass index, insulin resistance, and androgen levels were assessed.

**RESULTS:**

Women with polycystic ovary syndrome exhibited significantly lower epiregulin levels compared to controls (p<0.001). Epiregulin levels showed a negative correlation with polycystic ovary syndrome status and were inversely associated with body mass index, waist circumference, Homeostatic Model Assessment Insulin Resistance, triglycerides, fasting insulin, fasting glucose, and testosterone.

**CONCLUSION:**

Reduced epiregulin levels may contribute to the pathophysiology of polycystic ovary syndrome, highlighting its potential as a biomarker and therapeutic target. Further studies are needed to explore the underlying mechanisms and clinical implications.

## INTRODUCTION

Polycystic ovarian syndrome (PCOS), also referred to as ovarian hyperandrogenemia, is the most prevalent endocrine disorder affecting women of reproductive age. Its prevalence ranges from 6 to 8%, varying based on the diagnostic criteria applied^1–5^. PCOS is characterized by menstrual irregularities such as oligo-amenorrhea and dysfunctional uterine bleeding, symptoms of hyperandrogenism including hirsutism, acne, oily skin, and androgenic alopecia, as well as infertility^3–10^.

Epiregulin (EPI), a ligand of the epidermal growth factor (EGF) family, interacts with receptors in the ErbB family, initiating receptor dimerization and tyrosine phosphorylation. This interaction activates various intracellular pathways responsible for processes such as development, differentiation, apoptosis, adhesion, and migration^11–13^. EPI has also been implicated in the pathogenesis of multiple cancers^13,14^.

While the direct relationship between EPI and PCOS remains underexplored, some evidence suggests that other EGF family members may play a role in PCOS pathology^15^. EGF-like factors, including betacellulin, amphiregulin, and epiregulin, are known to facilitate oocyte maturation through autocrine and paracrine mechanisms^16–18^. However, the precise physiological connection between these factors and PCOS remains unclear.

Notably, EPI is involved in reproductive mechanisms, including ovulation and oocyte maturation. Given that disruptions in EGF signaling have been implicated in PCOS pathogenesis, this study aims to investigate EPI levels in patients with PCOS to elucidate its potential role in the pathophysiology of the disorder, offering insights into its underlying mechanisms and possible therapeutic implications.

## METHODS

### Ethical approval

This study was conducted in accordance with the principles outlined in the Declaration of Helsinki. Ethical approval for the study protocol was obtained from the Pamukkale University Non-Interventional Clinical Research Ethics Committee on August 4, 2015, under approval number 60116787-020/20820.

### Participants

The study included 67 participants, consisting of 39 women diagnosed with PCOS and 28 healthy controls, recruited from the Endocrinology and Metabolic Diseases Clinic at the Faculty of Medicine, Pamukkale University, Denizli, Türkiye. PCOS diagnosis was based on the Rotterdam 2003 criteria, which include:

Patients included in the study were required to meet criteria such as elevated serum total and free testosterone levels, oligomenorrhea (intervals exceeding 45 days between menses or fewer than eight menses per year), clinical hyperandrogenism, including acne, hirsutism, androgenic alopecia, or acanthosis nigricans, and ultrasonographic evidence of polycystic ovaries (≥12 follicles measuring 2–9 mm in diameter and/or ovarian volume >10 mL).

Participants with a history of cancer treatments, steroid use, thyroid dysfunction, neuroleptic or antidepressant use, or any hematological, renal, liver, or gastrointestinal diseases were excluded. Serum EPI levels were measured and compared across groups.

### Anthropometric evaluations

Anthropometric assessments included measurements of height, weight, waist circumference (WC), and body composition. BMI was calculated by dividing weight (kg) by height squared (m²), with obesity defined as a BMI >30 kg/m². WC was measured at the midpoint between the lowest rib and the iliac crest while participants stood upright. Total body fat mass, body fat percentage, and trunk fat mass were assessed using bioelectrical impedance analysis (BIA) with the TANITA BC-418 device (Tanita Corp., Tokyo).

Blood pressure was recorded after 5 min of rest in the supine position using an aneroid sphygmomanometer, and the average of three consecutive measurements was used. Lipid profiles and fasting blood glucose were assessed using venous blood samples collected after overnight fasting. Hormone and ferritin levels were measured from blood samples obtained between 9:00 and 10:00 AM.

### Biochemical analysis

Biochemical assessments included ferritin, triglycerides, fasting glucose, total cholesterol, and high-density lipoprotein (HDL) cholesterol levels. Fasting insulin levels were quantified using chemiluminescent immunoassays, while serum hormone levels were determined using electrochemiluminescence immunoassay.

### Epiregulin analysis

All participants provided informed consent prior to inclusion in the study. Serum EPI levels were measured using the Human Epiregulin ELISA kit (YHB1120HU).

### Statistical analysis

Data were analyzed using SPSS version 20.0. Levene's test was applied to evaluate the homogeneity of variances. Comparisons of EPI levels between groups were conducted using Student's t-test. Correlation analyses were performed to assess associations between PCOS and EPI levels. A 95% confidence interval was applied, and p-values <0.05 were considered statistically significant.

## RESULTS

Age, total cholesterol, systolic blood pressure (SBP), high-density lipoprotein (HDL), and low-density lipoprotein (LDL) levels did not differ significantly between the PCOS and control groups (p>0.05). In contrast, triglyceride levels and diastolic blood pressure (DBP) were significantly higher in the PCOS group (p<0.05). Moreover, BMI, waist circumference, fasting glucose, Ferriman–Gallwey score (FGS), and Homeostatic Model Assessment Insulin Resistance (HOMA-IR) were markedly elevated in PCOS patients compared to controls (p<0.001) (Table 1).

**Table 1:** Demographic data, epiregulin levels, and hormonal profiles of the polycystic ovary syndrome group and the control group.

	Control (n=28) (mean±SD)	PCOS (n=39) (mean±SD)	p-value
Age (year)	20.31±3.08	22.17±4.83	NS
BMI (kg/m²)	21.00±2.26	24.92±5.71	<0.001
Waist circumference (cm)	72.00±5.36	82.48±11.80	<0.001
Fasting glucose (mg/dL)	76.37±7.72	90.07±6.97	<0.001
FGS	4.82±7.10	15.40±8.28	<0.001
Total cholesterol (mg/dL)	154.58±25.68	165.28±34.27	NS
HDL (mg/dL)	49.39±9.75	46.36±10.51	NS
LDL (mg/dL)	90.10±20.30	99.31±30.03	NS
Triglyceride (mg/dL)	74.17±28.94	97.89±43.37	<0.05
SBP (mmHg)	97.59±18.25	103.33±11.08	NS
DBP (mmHg)	66.21±6.21	70.77±9.83	<0.05
HOMA-IR	1.34±0.55	2.49±1.71	<0.001
Epiregulin (ng/mL)	162.75±74.20	103.59±78.52	<0.001
Insulin (μIU/mL)	7.04±2.46	11.11±7.25	<0.01
FSH (Mıu/mL)	5.76±2.12	5.58±1.72	NS
LH (mIU/L)	5.96±1.74	6.59±3.17	NS
Estrogen (pg/mL)	45.65±54.99	56.96±57.88	NS
Testosterone (ng/mL)	36.00±14.82	50.91±26.34	<0.01
SHBG (nmol/L)	45.32±22.77	36.42±39.76	NS
DHEAS (μg/dL)	273.48±111.99	268.05±113.59	NS
TSH (μIU/mL)	2.15±1.45	1.86±0.90	NS
Prolactin [ng/mL]	19.01±8.56	19.81±10.12	NS

Values expressed mean±standard deviation (SD), Student's t-test. BMI: body mass index; FGS: Ferriman–Gallwey hirsutism scores; HDL: high-density lipoprotein; LDL: low-density lipoprotein; SBP: systolic blood pressure; DBP: diastolic blood pressure. HOMA-IR: homeostasis model assessment of insulin resistance; FSH: follicle stimulating hormone; LH: luteinizing hormone: SHBG: sex hormone binding globulin, DHEAS: dehydroepiandrosterone sulfate; TSH: thyroid-stimulating hormone; PCOS: polycystic ovary syndrome; NS: not significant.

**Figure 1:**
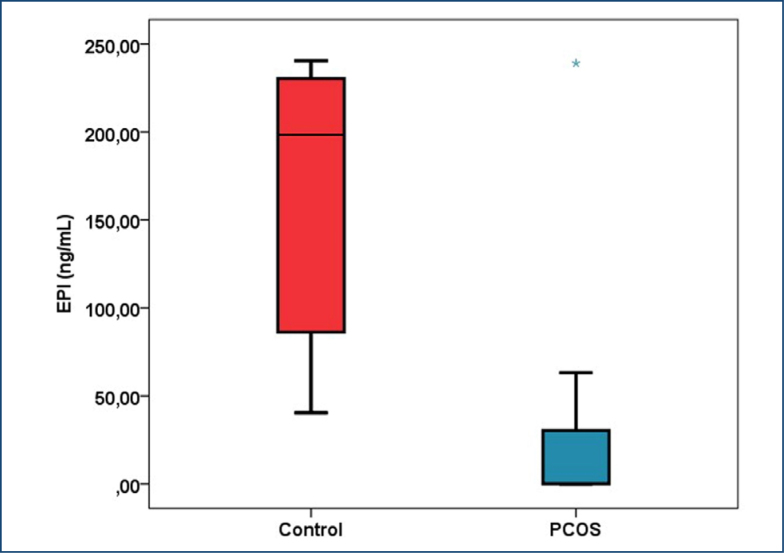
Statistical analysis of epiregulin levels in the patient and control groups, p<0.001.

A statistically significant reduction in serum EPI levels was observed in PCOS patients compared to controls, as illustrated in Table 1 and Figure 1. Additionally, insulin and testosterone levels were significantly elevated in PCOS patients (p<0.01). No significant differences were noted for other hormonal parameters (Table 1).

While no positive correlations were observed between EPI levels and any parameters, a significant negative correlation was identified between EPI and several clinical and biochemical parameters in PCOS patients. These parameters are detailed in Table 2.

**Table 2:** Parameters showing a significant correlation between epiregulin and polycystic ovary syndrome.

	Correlation	p-value
	**EPI**	
BMI (kg/m^2^)	-332**	<0.01
Waist circumference (cm)	-382**	<0.01
FGS	-564**	<0.01
HOMA-IR	-370**	<0.01
Fasting glucose (mg/dL)	-632**	<0.01
Triglyceride (mg/dL)	-328**	<0.01
Fasting insulin (mU/L)	-317**	<0.01
Testosterone (ug/L)	-253**	<0.01
	**PCOS**	
EPI	-791**	<0.01
BMI (kg/m^2^)	397**	<0.01
Waist circumference (cm)	485**	<0.01
FGS	650*	<0.05
HOMA-IR	393**	<0.01
Fasting glucose (mg/dL)	686**	<0.01
Triglyceride (mg/dL)	298*	<0.05
Fasting insulin (mU/L)	335**	<0.01
Testosterone (ug/L)	320**	<0.01
Diastolic blood pressure (mm Hg)	260**	<0.01

Pearson correlation,

* Correlation is significant at the 0.05 level;

** Correlation is significant at the 0.01 level;

(-): Negative correlation. EPI: epiregulin; BMI: body mass index; FGS: Ferriman–Gallwey hirsutism scores; HOMA-IR: homeostasis model assessment of insulin resistance; PCOS: polycystic ovary syndrome.

Correlations between PCOS and various patient parameters were further analyzed, with positively and negatively associated parameters presented in Table 2. These findings highlight the complex interplay between PCOS-related pathophysiology and patient characteristics.

## DISCUSSION

This study highlights a potentially significant link between EPI levels and PCOS, warranting further investigation. Our findings show that EPI levels are notably decreased in patients with PCOS, indicating a potential involvement of EPI in the pathogenesis of the condition. The observed negative correlations between EPI and key metabolic parameters, including BMI, waist circumference, fasting glucose, HOMA-IR, triglycerides, fasting insulin, and testosterone, support the hypothesis that EPI could be involved in the metabolic and hormonal disturbances associated with PCOS. These results are important as they provide new insights into the metabolic changes in PCOS and suggest that EPI might serve as a potential biomarker for evaluating disease severity or progression. Understanding the relationship between EPI and PCOS could lead to new therapeutic strategies and improve our ability to manage and treat this complex endocrine disorder.

When it comes to women of reproductive age, PCOS is the most common endocrinopathy and is often associated with ovarian hyperandrogenism. This condition affects a highly diverse patient population^19^. Clinical presentations, serum androgen levels, and ovarian morphology can vary significantly. The incidence of PCOS can differ based on diagnostic criteria but typically ranges from 6 to 8%^20,21^. PCOS is the most frequent cause of anovulatory infertility among women of reproductive age, with over 80% of affected women experiencing infertility^22–24^.

Epiregulin is a 46-amino acid protein and a member of the EGF family. This peptide hormone binds to the EGF receptor (EGFR/ErbB1) and ErbB4 (HER4) through ligand-dependent heterodimerization, subsequently activating signaling pathways involving ErbB2 (HER2/Neu) and ErbB3 (HER3). EPI plays diverse roles in both healthy and pathological conditions, including inflammation, cell proliferation, tissue repair, promoting angiogenesis, vascular remodeling, wound healing, and oocyte maturation^13^.

Dysregulated epiregulin activity has been linked to various cancers, including those of the colon, liver, breast, lung, bladder, stomach, head, and neck^13,25,26^. Consequently, EPI and components of the signaling network EGF/ErbB that contribute to its synthesis are potential targets for diagnostic and therapeutic interventions^13^.

Epiregulin's role extends to cellular processes such as growth, differentiation, and repair. By binding to the EGF receptor, it activates pathways that influence cell proliferation, migration, and survival and is involved in tissue regeneration^27,28^. Elevated levels of epiregulin are observed in response to tissue damage or inflammation. Abnormal epiregulin levels are associated with several diseases, including cancers and inflammatory conditions, making it a valuable subject of ongoing research to explore its potential as a biomarker and therapeutic target^13,25,26,28^. However, information on the relationship between EPI and PCOS remains limited.

EGF is found in the follicular fluid of the human ovary and acts on cumulus granulosa cells via the EGFR signaling pathway to control follicular development and oocyte meiotic maturation. It has been reported that epiregulin is effective as a meiotic stimulator for mural granulosa and theca cells^29^. Epiregulin might potentially be involved in the pathophysiology of PCOS. In the future, selective EGFR inhibition could provide a novel approach for treating PCOS^11,16,17,30,31^.

Moreover, EGF-like factors, including betacellulin, epiregulin, and amphiregulin, contribute to oocyte maturation through autocrine and paracrine pathways. However, their physiological roles in PCOS remain unclear. Research has shown that amphiregulin expression is downregulated in the ovarian follicular fluid, stromal cells, and cumulus cells of PCOS patients. The abnormal function of amphiregulin in PCOS and the reasons behind decreased amphiregulin levels in PCOS ovaries are still not fully understood.^15–17,32^.

The results of this investigation showed that PCOS patients had much lower EPI levels. Our findings also revealed a notable negative correlation between EPI and BMI, waist circumference, Ferriman–Gallwey hirsutism scores, HOMA-IR, fasting glucose, triglycerides, fasting insulin, and testosterone. Between PCOS patients and control volunteers, we examined EPI levels, anthropometric measurements, hormonal profiles, and several metabolic indicators. Other hormonal indicators did not significantly differ between the PCOS group and the other groups, despite the PCOS group having much higher levels of insulin and testosterone. These findings underscore the significance of considering both hormonal and metabolic variables in order to fully comprehend PCOS and point to possible metabolic disturbances linked to the illness.

The effects of EGFR pathways and EGF-like factors in PCOS remain unclear, indicating a need for further research. Future studies should aim to elucidate the mechanisms underlying the observed changes in EPI levels and explore their clinical implications. Additionally, investigating potential therapeutic strategies to modulate EPI levels could provide new approaches for managing PCOS and improving patient outcomes.

The potential role of EPI in PCOS suggests new avenues for clinical applications. Given the strong correlations between EPI and key metabolic and hormonal parameters, EPI could serve as a biomarker for assessing disease severity and metabolic disturbances in PCOS patients. Furthermore, therapeutic strategies targeting EPI-related pathways may offer novel treatment options for PCOS, particularly in managing metabolic dysfunction and hormonal imbalances. Selective modulation of EGFR signaling, including EPI, may provide a targeted approach to addressing the reproductive and metabolic challenges associated with PCOS, improving patient outcomes and quality of life.

This study has several strengths that contribute to its scientific value. First, it provides novel insights into the relationship between EPI and PCOS, adding to the limited body of literature on this topic. Second, the study evaluates multiple metabolic and hormonal parameters, allowing for a comprehensive assessment of EPI's potential role in PCOS pathogenesis. Third, the robust statistical analysis strengthens the validity of the observed correlations. Finally, by highlighting the involvement of EPI in both metabolic and reproductive aspects of PCOS, this study opens new avenues for potential therapeutic interventions, making it a valuable contribution to the field.

Despite the significant findings, this study has several limitations. First, the sample size was relatively small, which may limit the generalizability of our results. Larger, multicenter studies are necessary to validate our findings and ensure broader applicability. Second, this study relied on a cross-sectional design, which prevents us from establishing causality between EPI levels and PCOS. Longitudinal studies are needed to assess whether changes in EPI levels contribute to the progression of PCOS or result from the condition. Third, while we identified correlations between EPI and various metabolic and hormonal parameters, we did not investigate the underlying molecular mechanisms. Future studies should focus on elucidating the precise pathways through which EPI influences PCOS pathogenesis. Additionally, factors such as diet, lifestyle, and genetic predisposition were not accounted for in our analysis, which may have impacted our findings. Finally, the study population was limited to a specific demographic, and differences in ethnicity or genetic background may influence EPI levels and their role in PCOS. Further research incorporating diverse populations is necessary to establish more comprehensive conclusions.

This study demonstrates that EPI levels are significantly lower in PCOS patients and are negatively correlated with key metabolic and hormonal parameters. These findings suggest that EPI may play a role in the metabolic disturbances associated with PCOS and could serve as a potential biomarker for disease severity. Further research is needed to elucidate the underlying mechanisms and explore the therapeutic potential of targeting EPI pathways in PCOS management. Understanding the involvement of EPI in PCOS may contribute to the development of more effective diagnostic and treatment strategies for this complex disorder. Future studies focusing on larger populations and mechanistic pathways will be essential to confirm these findings and advance potential clinical applications.

## Data Availability

The datasets generated and/or analyzed during the current study are available from the corresponding author upon reasonable request.
